# Simulations of goaf surface subsidence via filling control

**DOI:** 10.1371/journal.pone.0261740

**Published:** 2022-01-21

**Authors:** Yong Yang, Minsi Zhang, Gaojian Hu, Kai Guan

**Affiliations:** 1 Engineering Research Center for Seismic Disaster Prevention and Engineering Geological Disaster Detection of Jiangxi Province, East China Univ. of Technology, Nanchang, China; 2 Key Laboratory of Rock Mechanics and Geohazards of Zhejiang Province, Shaoxing, Zhejiang, China; 3 School of Resources and Civil Engineering, Northeastern University, Shenyang, China; University of Science and Technology Beijing, CHINA

## Abstract

Xincheng Gold Mine is taken as an example to investigate the ground subsidence that results from filling. Both numerical simulations and simulation experiments are conducted to simulate the deformation process at the stope roof and bottom from excavation and filling. The assumption of macroscopic continuity from traditional continuum mechanics models is overcome. The simulation results demonstrate that the ground subsidence is slowed due to filling. The total trends of the top and bottom displacements are sinkable and upturned, respectively. Moreover, with an increased buried depth and lateral pressure coefficient, the displacements of the top and bottom of the stope increase as well. The characteristics and evolution of the displacement vector field of the rock mass are macroscopically and microscopically studied over the excavation progress. This provides technical support for stope safety production.

## Introduction

Roof damage and surface subsidence caused by underground mining have been among the most difficult problems in mining technologies. Surface subsidence is a widespread problem that is frequently caused by underground mining [1]. Filling mining technologies are among the most effective ways to ensure safe and efficient ore recovery and solve technical problems such as residual ore recovery, deep ground pressure control, and goaf stability [2]. To date, treatment methods for goafs are based primarily on filling. The stress distribution of the surrounding rock can change based on the interactions of the backfill and goaf surrounding rock, which can prevent surface collapse. With an enhanced awareness of the resources and environmental protection, the demands on the stability, fluidity, and plasticity of the filling slurry are increasing. Therefore, high-concentration filling technologies will become the development direction of goaf governance research.

Goafs formed during mineral mining are prone to collapse due to unbalanced stresses, suggesting they should be filled to reduce this possibility. To date, the data indicate that filling mining has accounted for more than 50% of China’s metal mining. Since the 1960s, filling mining has been widely used in non-coal mining throughout China, and research on filling mining has been given increased attention by experts and scholars [3,4]. The FLAC 3D numerical simulation software was used to study the ground surface deformations caused by cemented filling in the Jinchuan No. 2 mining district. The effects of the elastic modulus of the filling body on the surface subsidence had previously been considered [5]. To improve the control effects of surface subsidence caused by backfilling mining, Qu et al. estimated the subsidence factor for full goaf backfilling and analyzed how these factors affect the surface subsidence through theoretical analyses and numerical calculations [6]. To reveal the surface deformation law and internal mechanism induced by filling mining under complex environments of high tectonic stress, Jiang et al. emphasized the space-time evolution process for surface deformation and subsidence [7]. To study the strength behaviors of solid backfill materials, Zhan et al. developed a method to generate random gravel models using computational geometry algorithms. Based on these, several biaxial tests under different confining stresses were conducted using the Particle Flow Code (PFC2D) [8]. The built-in FISH language of FLAC 3D was used by Meng et al. for a nonlinear compaction program of a backfill body, and the overlying strata movement was accurately simulated after the gob was filled with gangue [9].

The quantitative evaluation results of the stability of the goaf as affected by waste rock filling indicated the necessity to conduct ground pressure monitoring while filling the goaf [10]. The stability of the open slope and goaf under unfilled limestone soil and tailings was simulated and analyzed by Rui et al. [11]. The monitoring data for ground deformation and macroscopic damage features of the mining area were analyzed by Xia et al. for the Chengchao Iron Mine [12]. Overlying strata movement of recovering standing pillars with solid backfilling was studied by the physical simulation in 2016 [13]. A comprehensive analysis was proposed and used to analyze the surface subsidence characteristics and damage range by Zhao [14]. The GPS monitoring results showed that the vertical displacement reached 40.3 mm at the JC5 monitoring station and was accompanied by ground crack development. Ground deformation in the vertical and horizontal directions was studied by Dong [15]. The two-dimensional ground deformation monitoring was performed in Shanghai. The vertical deformation rates decreased to less than 1 cm/year in most districts of the Shanghai area.

To date, while there are several methods for mining research that have been developed, the relationship between surface deformations and strata movement is still unknown, especially considering that the engineering background and surrounding environments vary for different mines. It is generally considered that problems of rock mass movement and surface displacement in metal mines, which are mined using the backfill mining method, are not significant. However, this is not true and there appears to be more significant rock mass movement and surface deformation phenomenon in several metal mines. Thus, it is important to address this problem.

Several research methods, such as in-situ monitoring, numerical simulations, laboratory testing, and theoretical analysis, are widely used in practice and play an important role in promoting scientific research in relevant engineering fields [16]. Thus, mining technologies have been greatly improved. While various mine monitoring technologies for rock strata movement and surface ground settlement have become more accurate and efficient, the associated research still needs to be better developed. This is key to ensure the sustainable development of the mining economy.

As the finite element method is used primarily to solve continuum problems under the premise of small deformations, it has some limitations to solve large deformations and nonlinear problems of the rock mass. Here, the PFC2D, which is a two-dimensional discrete element operator method generated from irregular particles and particle contact interfaces, is used to monitor changes in the displacement field for the middle section of Shandong gold mine under the influence of excavation and filling [17,18]. A similar simulation experimental method is used to study the displacement field deformations of the roof and bottom. The characteristics and evolution of the rock mass displacement vector field during cavitation are studied from both macroscopic and microscopic perspectives. In the process of the metal mining, the protection against surface subsidence becomes very important. It is of great significance to improve the safety of mining through various detection and protection. This provides technical support for cavitation safety production.

## Influencing factors of surface subsidence in filling mining

The influencing factors of filling mining on the surface subsidence include the filling rate and compression rate of the backfill. The filling rate of backfill is given by

$$r=\frac{\upsilon_{1}}{\upsilon }$$ (1)

where *υ*_1_ is the volume of filling material, and *^v^υ* is the produced rock volume. One of the most efficient and direct ways to slow surface mining and subsidence is to improve the filling rate of the backfill [19]. Theoretically, if the filling rate is close to 1, the surface subsidence can be completely controlled through filling mining. Due to the deformation of the surrounding rock in the goaf before filling, limitations of filling technologies make it impossible to fill materials with complex shapes into the goaf or realize goaf filling and roof connection. In actual filling processes, the filling rate is less than 1. However, under the same filling material, filling technology, and mining geological conditions, a larger filling rate gives a better mining subsidence control effect.

The compression rate of the backfill is given by

$$S=\frac{l_{1}}{l}$$ (2)

where *l*_1_ is the maximum compression subsidence, and *l* is the height before compression. Under the action of dead weight and overlying strata, the backfill produces compression deformation and settlement. If the subsidence caused by compression backfill deformation in the goaf is too large, the mining subsidence becomes out of control.

## Analysis of surface subsidence in goaf under filling control

The metal mining process consists of rock breakage and instability control. Three laws should be followed during mining: the laws of ground pressure, rock caving, and loose mass flow. The main purpose of monitoring deformations of the roof and bottom is to determine variations in the deformation law for the goaf with time or other factors in addition to the movement law of the ground pressure. This is also used to analyze and evaluate the stability of the goaf based on the deformation law of the displacement field for the roof and bottom.

### Geological background

An ore deposit in the western margin of the Jiaobei uplift in the middle section of Xincheng gold mine in Shandong province is selected as the engineering background. Shandong Xincheng Gold Mine is located in Jincheng Town, Laizhou City, Shandong Province, China. The regional tectonic activity is relatively weak, and the shallow quaternary unconsolidated rock layer is thin. The bedrock is solid magmatic rock and metamorphic rock, which has a high hardness and mechanical strength. The rock quality is considered good and excellent, and the rock mass is relatively intact and has good stability.

**Fig 1:**
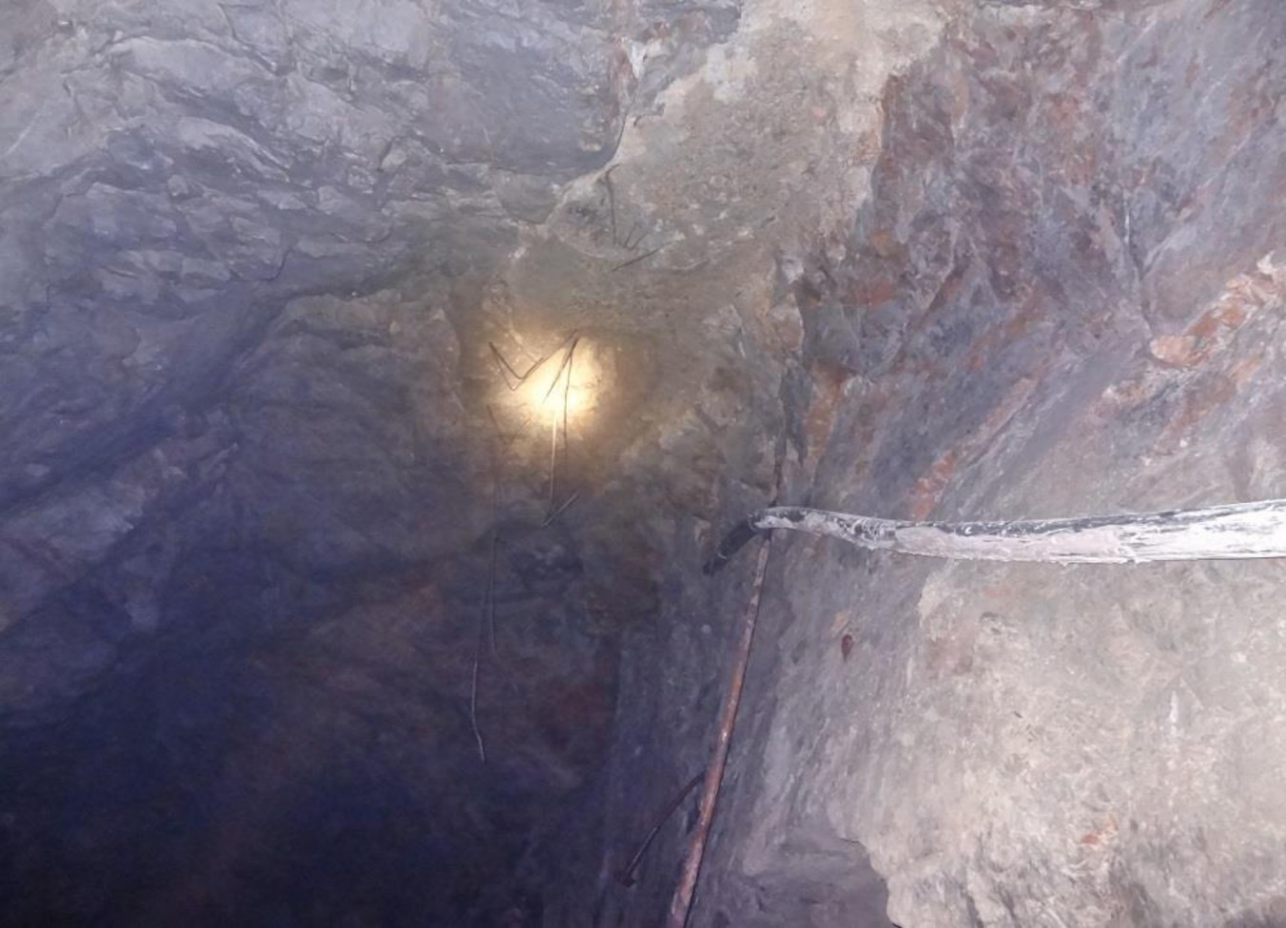
Photograph of the stope roof.

The lithology is serinite granodiorite, which has a high mechanical strength but with relatively developed fractures. The rock mass is cut into complex structural patterns of different sizes using crisscrossing fractures. In general, cracks can be closed in many small ways. Though different shapes of rocks are inlaid with each other, they still maintain a good stability. Therefore, most tunnels do not require support. It is noted that part of the larger cracks often has mud and slight slips can be caused. Roof collapse is readily caused in intersections, especially for inverted wedge roof formations, and the maximum height is more than 3 m. The quality of the artificial roof is poor and the rebar is not evenly arranged and does not bond well with the concrete (Fig 1). Therefore, more attention should be given to the roof stability.

The ore body, roof, and bottom rocks consist of hard and semi-hard rocks. The rock mass is stable, and its engineering geological conditions are simple. The rock stratum is poor in the local fracture zone or under altered rock engineering geological conditions. Only the main fracture surface of Jiaojia in the region needs shotcrete and consolidation. The remaining areas do not require support, indicating they can be safely mined. Rock samples were selected from different parts of the site to carry out the indoor rock point load test. The mechanical parameters of the rock blocks were obtained. Then the point load strength, the tensile strength and the compressive strength of rock sample is calculated. The macroscopic mechanical parameters of the rock mass are calculated by the Hoek-Brown method.

$$\sigma_{1}=\sigma_{3}+\sigma_{ci}{\left(m_{b}\frac{\sigma_{3}}{\sigma_{ci}}+s\right)}^{\alpha }$$ (3)

where *σ*_1_ and *σ*_3_ are the maximum and minimum principal stress of the failure rock mass, *σ*_*ci*_ is the uniaxial compressive strength of the complete rock blocks, *m*_*b*_ is the Hoek-Brown constants, *s* and *α* are the material parameters of the rock mass which depend on characteristics of the rock mass. The formulas of equivalent elastic modulus, the compressive strength, the tensile strength, the friction Angle, the cohesion and other parameters of the rock mass are no longer listed one by one. The calculated rock mass parameters are shown in Table 1.

**Table 1:** Macro parameters of the rock mass.

	Elastic modulus/GPa	Compressive strength/MPa	Tensile strength/MPa	Cohesion/MPa	Poisson’s ratio	Friction angle	The material parameter	
							*α*	s
**Rock mass**	15.1~17.2	16.5~20	0.67~0.71	1.5~1.8	0.26	0.5	0.501	0.0067

## Establishment of numerical model and parameter selection

The PFC2D approach models the movements and interactions of circular particles based on the discrete element method (DEM). The constitutive behavior of a material is simulated with the PFC by associating a contact model with each contact. It is also capable of modeling a brittle solid by bonding each particle to its neighbors. The resulting assembly is regarded as a “solid” that has elastic properties and is capable of “fracturing” when bonds break progressively. The PFC2D contains extensive logic to help model solids as close-packed assemblies of bonded particles. Considering the accuracy and number of calculations, this section selects the normal distribution of the sample particle with a radius that varies from *R*_max_ to *R*_min_.

The in-situ stress of the V# middle section in Xincheng gold mine is obtained using the in-situ stress regression calculations. This indicated that the maximum principal stress direction is perpendicular to the orebody strike direction. The field investigation showed that the deformation of the roadway, which is oriented along the direction of the ore body, is relatively large and the resulting support pressure is high during mining. Therefore, the direction of the stope is designed to be parallel to the square of the ground stress.

**Fig 2:**
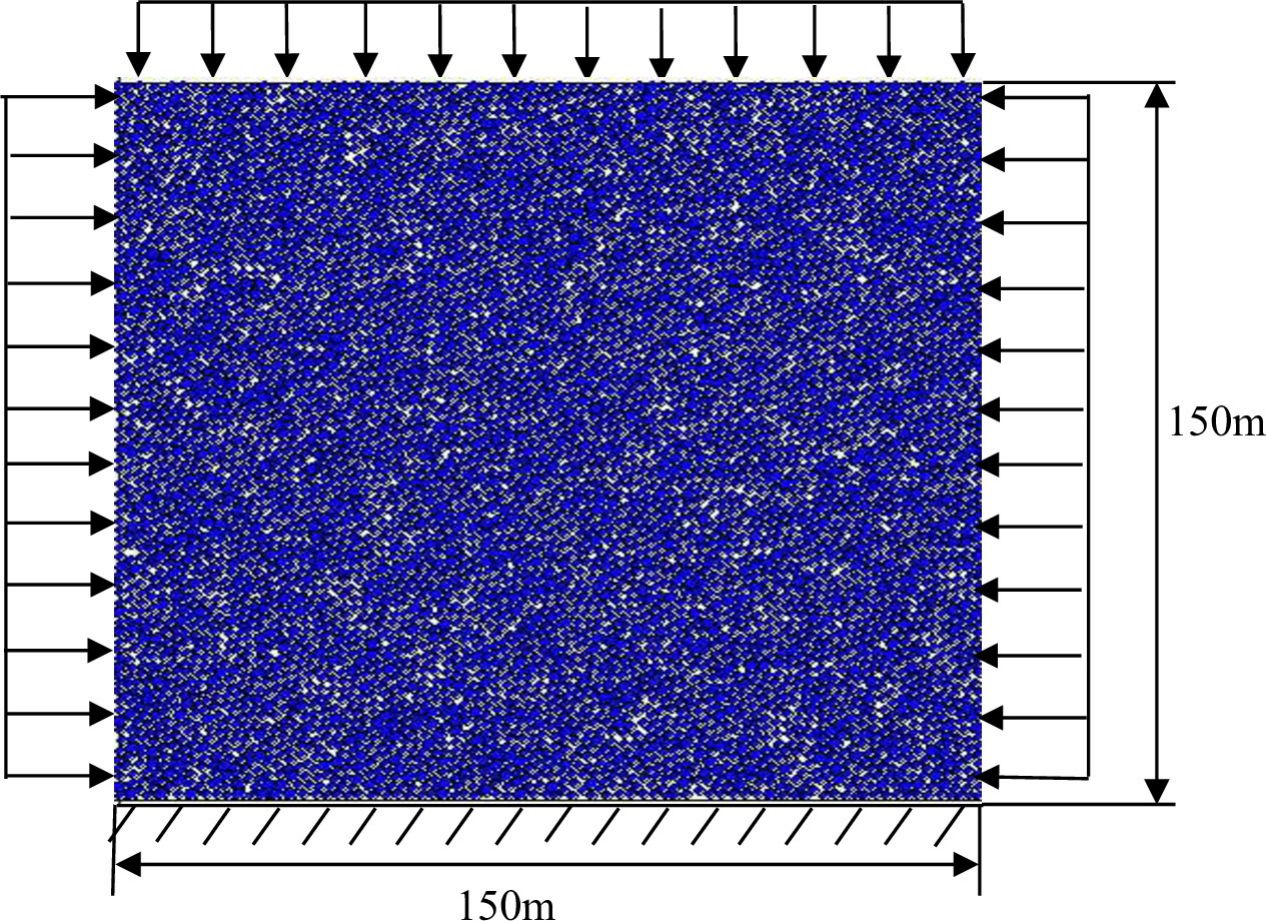
Illustration of the PFC numerical model.

The particle model diagram is shown in Fig 2. In the PFC, numerical experiments are generally required to calibrate the micro-parameters. However, the calibration process is complex, and it is necessary to control the method to quickly complete the calibration. Here, the uniaxial and biaxial numerical tests of the rock materials were established through the PFC2D. The basic physical meanings of the bond and contact models in the DEM in conjunction with the conventional splitting strength, shear strength, and modulus of rock materials were used to determine the relationship between the microscopic parameters of the discrete elements and the macroscopic performance parameters, such as material modulus and strength. In addition, the parameter calibration process related to parallel bonding was studied. The relationship between the microscopic and macroscopic parameters is summarized, and the mechanical parameters of the considered materials are determined.

The parameters were adjusted through the indoor uniaxial compression tests of the rock samples, which are given in Table 2. The size of the model is set as 150 m × 150 m. A 20 mm square cavity was excavated in the horizontal direction of the specimen to simulate an underground cavity. The boundaries of the model are provided with walls. The force exerted on the boundary acts directly on the wall and then transmits to the particles. The stress in the X-direction is applied to the left and right boundaries of the model to simulate the actual stress state of the rock mass. The displacement in the Y-direction is constrained at the bottom of the model, and a stress boundary is applied at the top. The stress load is uniformly distributed to simulate the overlying rock mass with different burial depths. Gravity is not considered. Several simulations were performed for the above structural parameters. Different lateral pressure coefficients (*λ* = 1, 2, and 3) and burial depths (H = 700, 900, and 1100 m) were considered to simulate the stability of the stope after cavity treatment.

**Table 2:** Parameters of the rock sample.

Parameters	M
**The minimum radius */m***	0.15
**The connection modulus of particles/*GPa***	49
**The connection strength of particles / MPa**	50
**The connection shear strength of particles / MPa**	49
**The friction angle**	0.5
**The elasticity modulus / MPa**	48
***μ***	0.15
***b_kn /b_ks***	4.0

The backfill materials and filling ratio were selected based on the requirements of the backfill and compression rate of the filling ratio. The backfill materials and their ratio included a filling aggregate of desliming-graded tailings with a sludge content of less than 15%. The cementing material was 325# ordinary Portland cement, and the gray-sand ratio was 1:15. Some mechanical parameters of the backfill are given in Table 3.

**Table 3:** Laboratory test properties of the backfill.

Parameters	M
**The volume-weight / *t•m*** ^***-3***^	0.5
**Uniaxial compressive strength / MPa**	0.1
**The elasticity modulus / MPa**	54.2
**The cohesive force / MPa**	0.02
**Poisson’s ratio**	0.17
**The friction angle**	20

### Analysis of goaf surface subsidence

**Fig 3:**
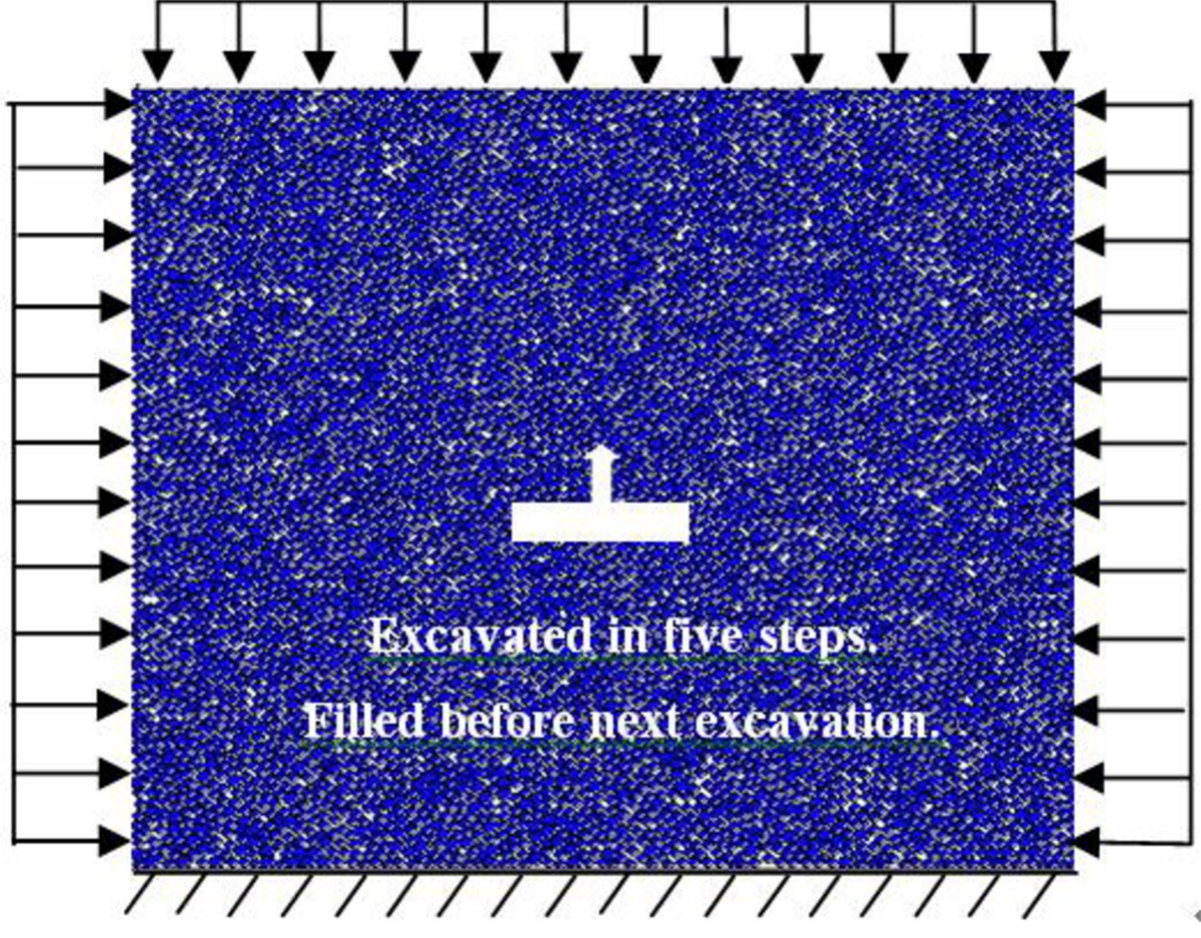
Illustration of the numerical model.

**Fig 4:**
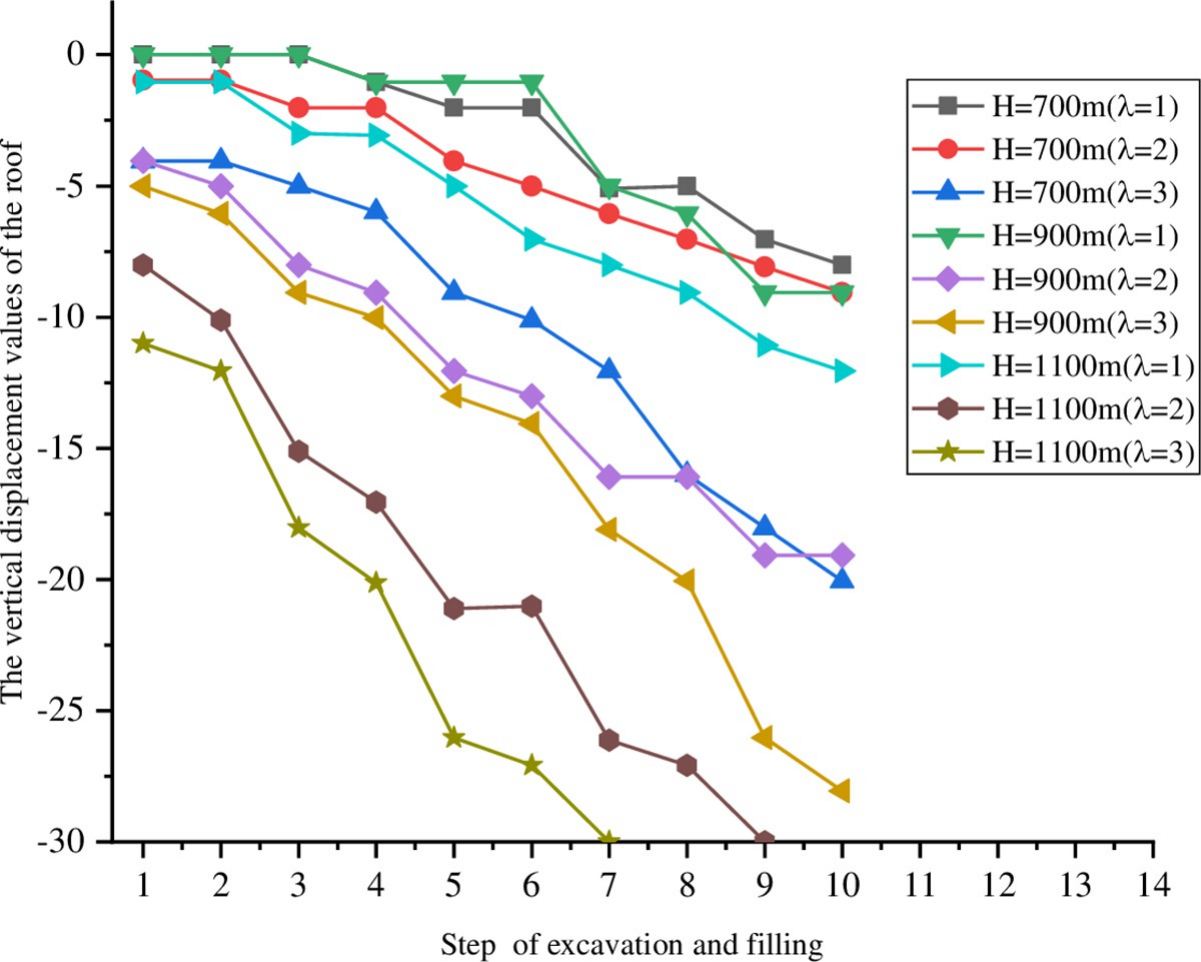
Vertical displacement of the monitoring points on the roof at different depths.

**Fig 5:**
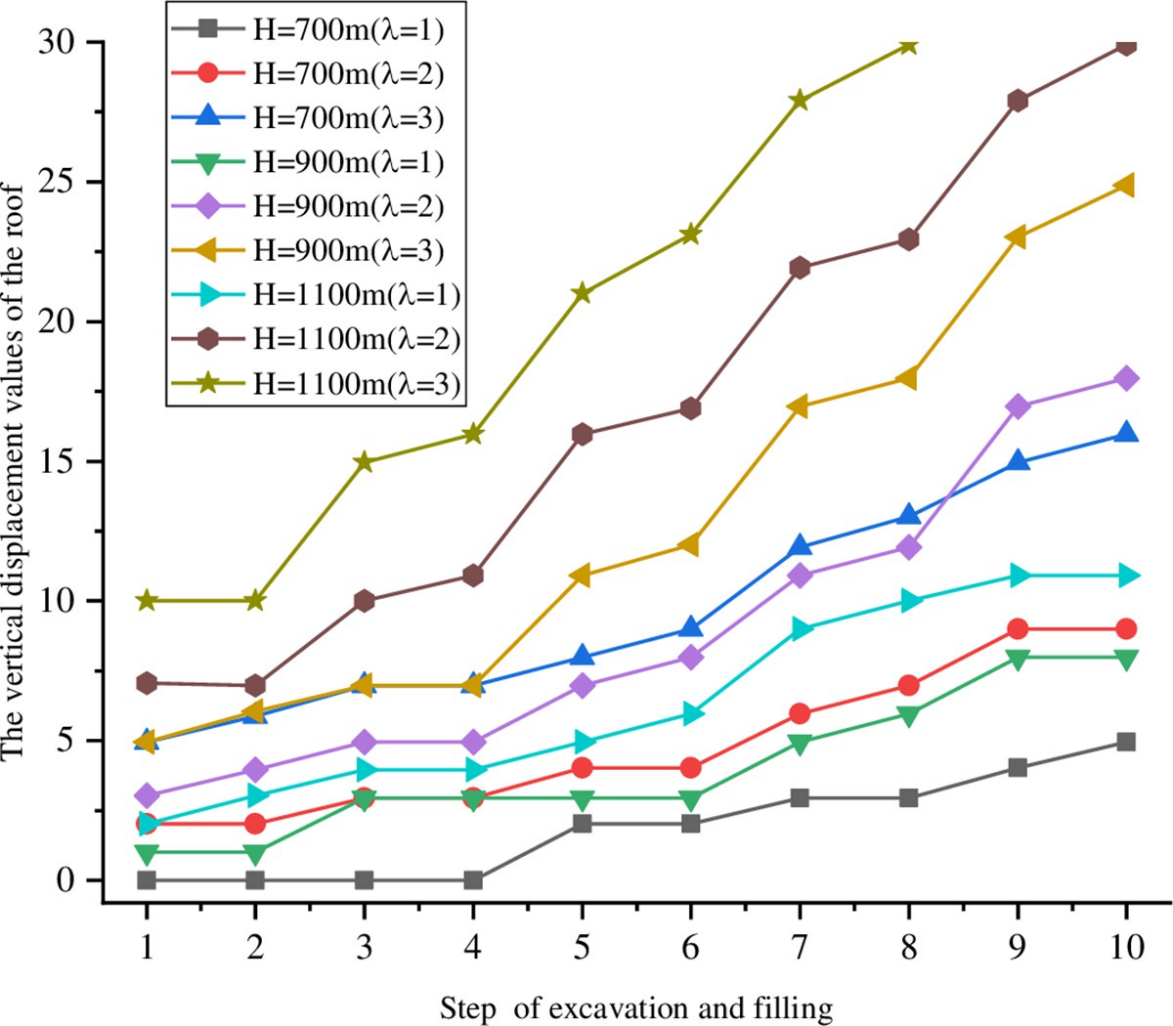
Vertical displacement of the monitoring points at the bottom at different depths.

The goaf was excavated in five steps with a span that is fixed for 20 m. The excavations were 10 m high each time. After the excavation was completed each step, it was filled before the following excavation. The lateral pressure coefficients were fixed at *σ*_*x*_ = *λσ*_*y*_ and *λ* = 1, 2, and 3. At the same time, different burial depths of H = 700, 900, and 1100 m were considered. The excavation model diagram is shown in Fig 3. The vertical displacements of the monitoring points on the roof and bottom under different excavation filling steps for various buried depths and lateral pressure coefficients are shown in Figs 4 and 5.

The roof and floor displacement deformations were small and there was no damage around the stope when the lateral pressure coefficient was 1 under different buried depths. With the advancement of the excavation and filling step-by-step, the displacement of the roof and floor under different buried depths reached approximately 10 mm after the fifth excavation. This has the risks of instability and collapse. The displacement deformations of the roof and bottom increased and there was damage around the stope when the lateral pressure coefficient was 2. The displacement of the roof and floor reached 9 mm at a buried depth H = 700 m, compared with 19 mm at H = 900 m and 30 mm at H = 1100 m. The displacements of the roof and bottom changed gradually when H = 1100 m and λ = 3, which reached 30 mm at the fourth excavation. The stability of the stope from the numerical simulations does not accurately follow reality. Instead, it can only be concluded that the rock mass around the cavitation tends to be increasingly unstable as the exposed area of the stope grows.

In the excavation and filling processes, the overall trend of the roof displacement is to sink, while the overall trend of bottom displacement is upward. With larger buried depths and lateral pressure coefficients, the displacements of the roof and bottom increase. In addition, changes in the displacement indicate that although the backfill does not change the subsidence of the roof or upward trend of the bottom, it has a certain influence on the stope stability. This slows down changes in the roof and bottom displacements and controls the subsidence of the ground. Based on the deformation characteristics of both the roof and bottom of the goaf, a point column support can be adopted in unstable areas.

## Similar simulating experiment for filling control settlement

### Similar material simulating experiment

**Fig 6:**
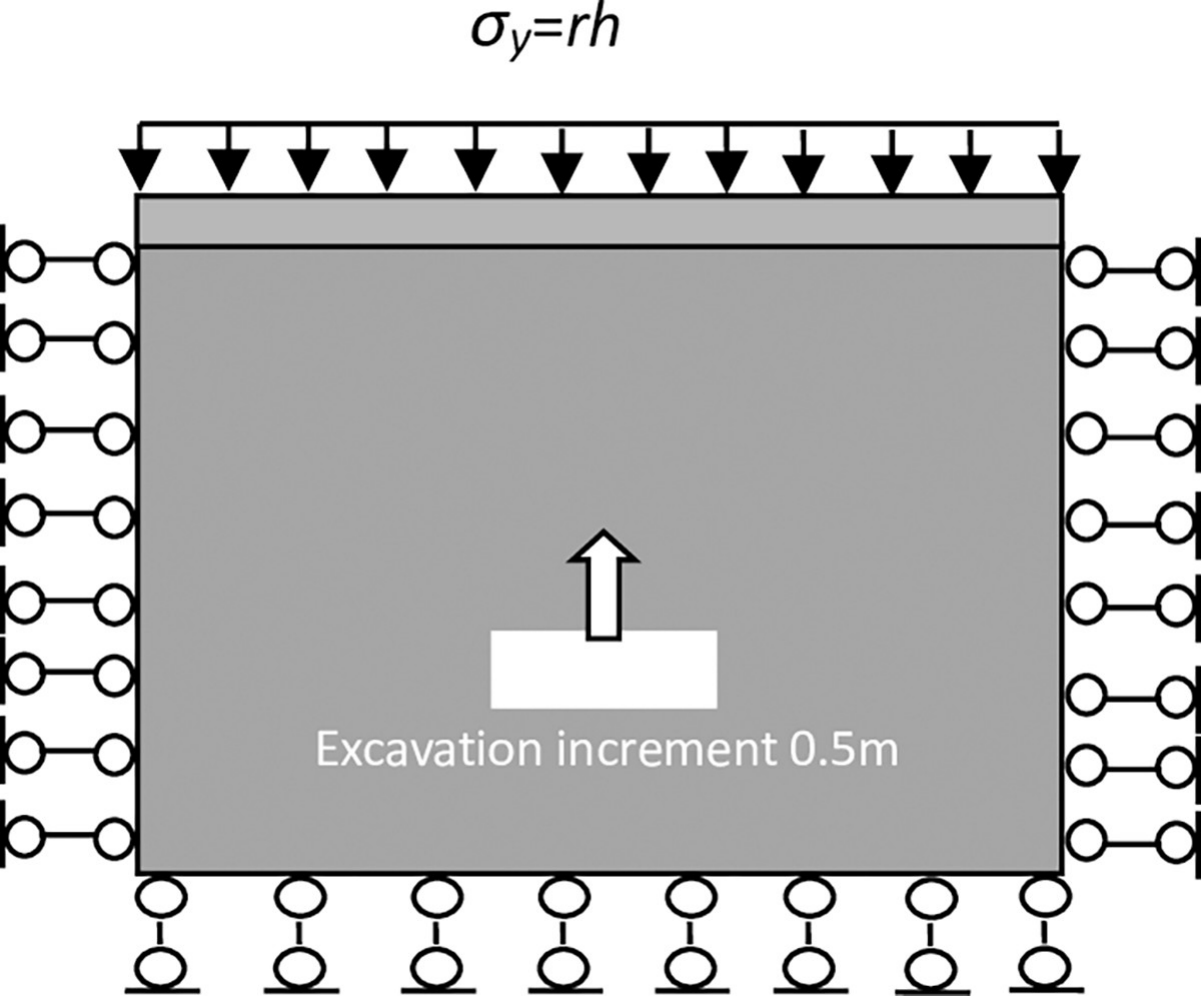
Layout of the experiment.

In the similar simulation experiment, the device is 5 m long, 2 m wide, and 5 m high and is surrounded by steel plates. The boundary in the model is set to be constrained. At a height of 1 m, the fixed span was 1 m, and the excavation was carried out in 5 steps with a height of 0.5 m each time. After each step, the filling was filled first and the next excavation was performed. The lateral pressure coefficient is taken to be 1, 2, and 3, and the displacement field variations of the roof under 700, 900, and 1100 m working conditions are simulated. The model plane diagram is shown in Fig 6.

**Fig 7:**
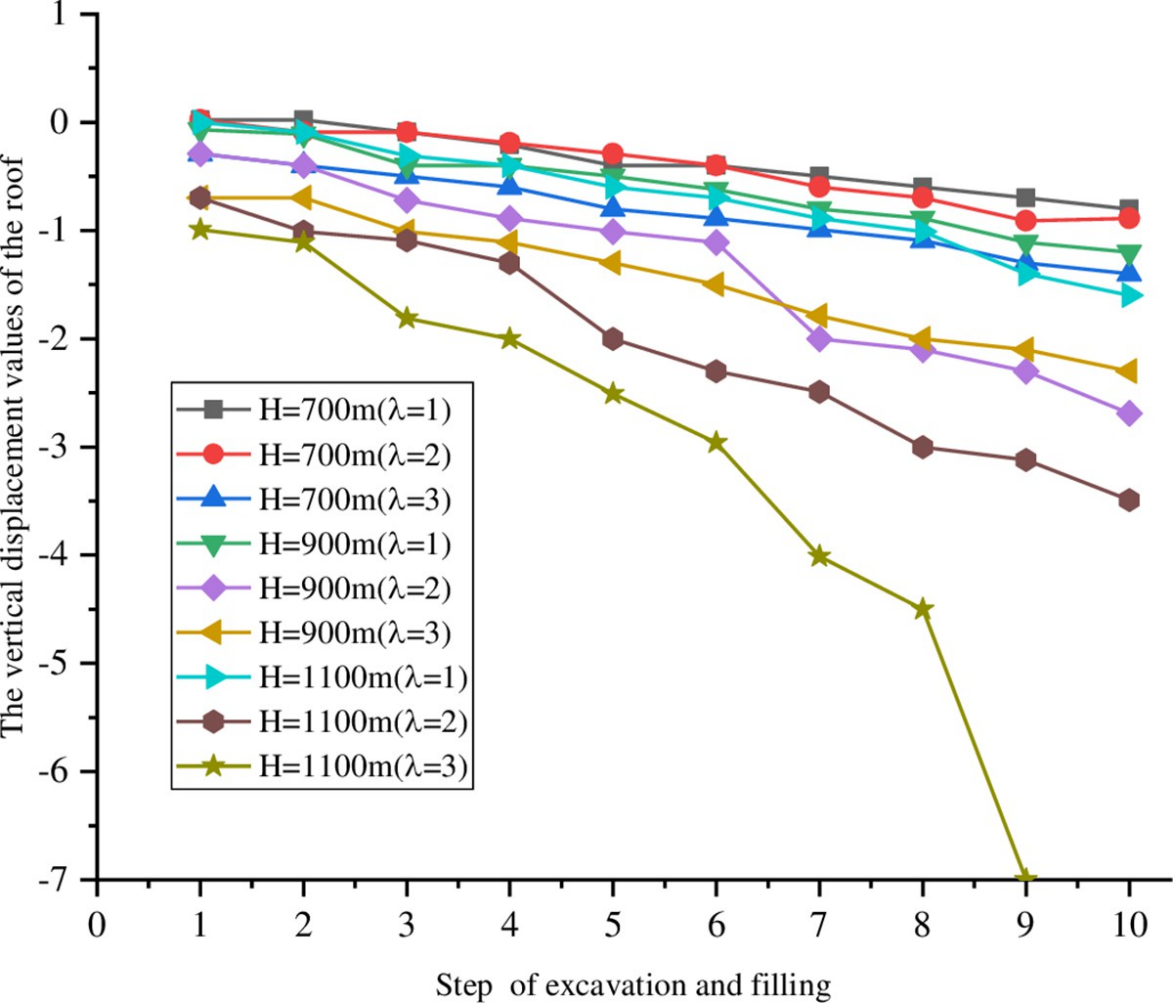
Vertical displacement of the monitoring point on the roof at different depths.

**Fig 8:**
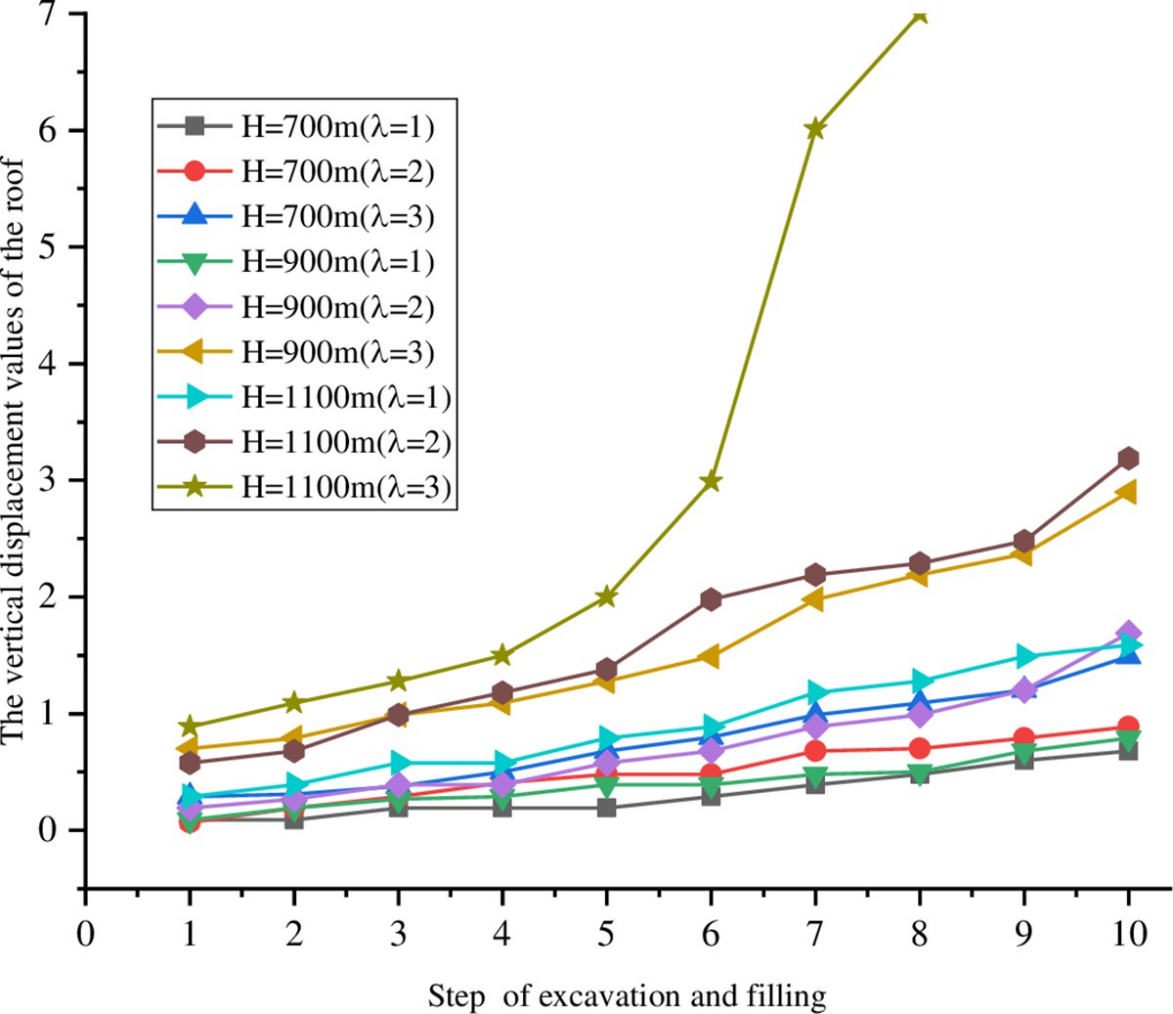
Vertical displacement of monitoring points on the bottom at different depths.

The small scale of the model causes the boundary effect. The influence of the factors besides the control variables on the test results was excluded as much as possible by setting the initial conditions of the same control area and the research object. To make the finally results comparable to the numerical simulations, the backfill and its ratio used in the test was similar to that from the numerical simulations based on similarity theory. The vertical displacement of the top and bottom monitoring points under different excavation filling steps, buried depths, and side pressure coefficients are shown in Figs 7 and 8.

### Simulation results and analysis

The numerical simulations show similar overall downward trends for the roof displacement, while the overall trend of the bottom is upwards in the excavation and filling processes with different buried depths and side pressure coefficients. When the buried depth is above 1100 m, the displacement of the roof and the bottom increases with the lateral pressure coefficient. When λ = 3, the resulting displacement change exceeds the safe value range.

The displacements of the roof and bottom are close to 1 mm after excavation in the fifth step when the buried depth is 700 and 900 m and the lateral pressure coefficient λ is 1 or 2. Thus, there is risk of instability and collapse for the stope. The displacements of the roof and the bottom are close to 1.5 or 2.5 mm after excavation in the third step when the lateral pressure coefficient λ is 3. There is a local damage point in the stope, and the displacements of the roof and the bottom reach or approach 1 mm after excavation in the fourth or fifth steps when the buried depth is 1100 m and the lateral pressure coefficient λ is 1. Thus, the stope has the risk of instability and collapse. The roof displacement reaches 1 mm after excavation in the second step when the lateral pressure coefficient λ is 2. This gives the risk of instability and collapse for the rock mass stope.

In short, increases in the lateral pressure coefficient cause the rock mass of the stope to be increasingly unstable. However, changes in the displacement of the roof and the bottom slow from the presence of the backfill, which controls the subsidence of the surface. Compared with the goaf surface subsidence on-site and the numerical simulations, the results are conservative and support measures should be taken in the weak area.

## Discussion

The above analysis shows that the surface is gradually affected by the movement and instability of the rock mass. The magnitudes of the surface ground movement are related primarily to the mining depth, mining thickness, and mining speed of the goaf. In the excavation and filling processes, the stress-strain relationship exhibits a multi-term variation trend, and the strain increases non-linearly with the stress. The stress-strain relationship is not proportional but still presents a one-to-one correspondence.

**Fig 9:**
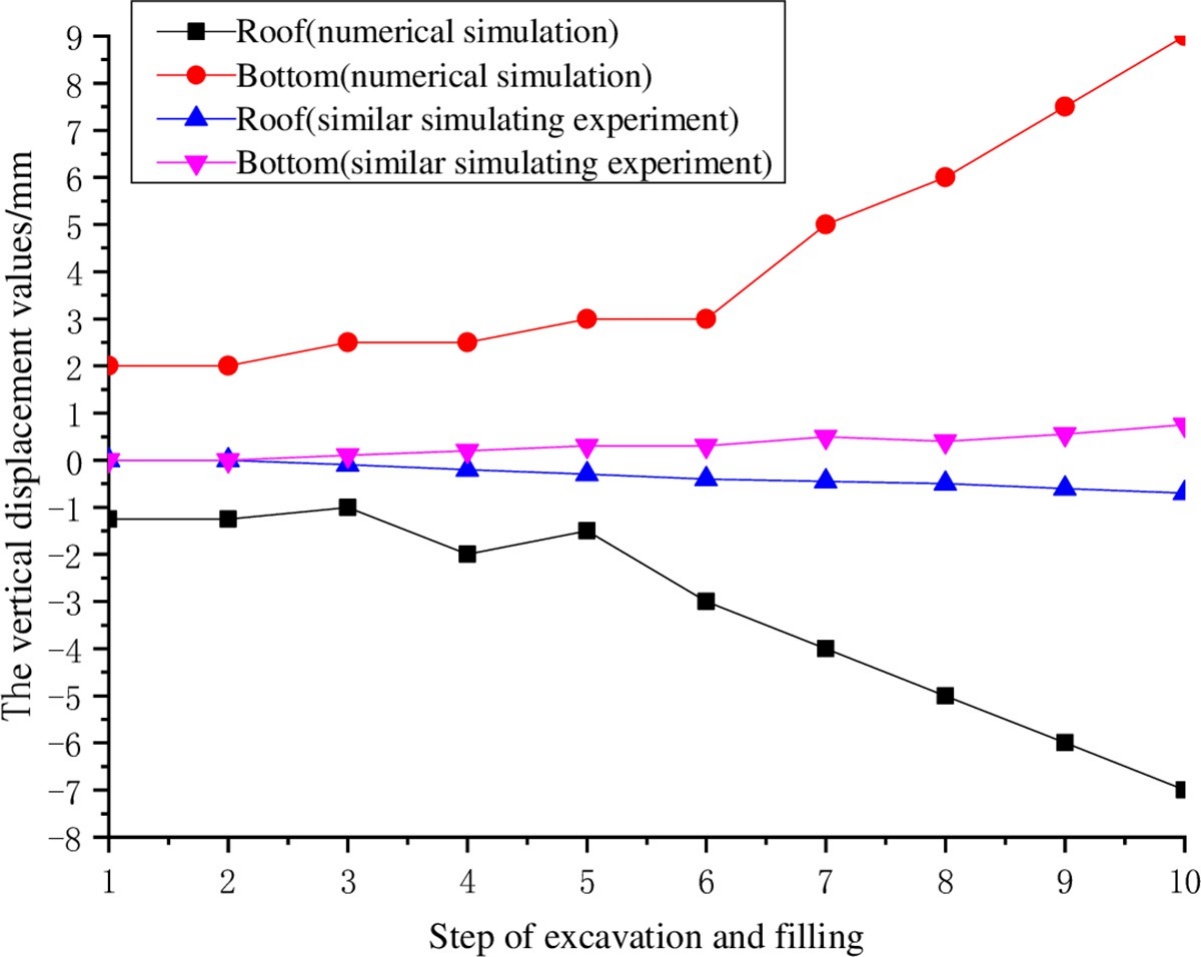
Comparison between the numerical simulations and similar simulation experimental results (H = 700, λ = 2).

A buried depth of 700 m and lateral pressure coefficient λ of 2 are taken as an example, as shown in Fig 9. With an increasing number of excavation steps, the deformation is slow at first and then enters a rapid deformation for the stage. In general, the stresses caused by mining subsidence and landforms are superimposed along the same direction and the deformation of the roof is greater than at the bottom. Compared with the numerical simulations, the results of the similar material simulating experiment are more conservative with a maximum displacement below 1 mm. The maximum displacement of the numerical simulation results only reaches up to 9 mm. This indicates that when the numerical simulations are adopted to estimate the actual situations under these working conditions, the safety factor should be set slightly smaller, which can be close to reality.

## Conclusion

The influence of backfill on the surface subsidence of the stope is analyzed using the similar simulation experiment method and numerical simulations. The characteristics and evolution of the displacement vector field of the stope roof and bottom during rock excavation are studied from both macroscopic and microscopic perspectives. This provides technical support for stope safety production. The following conclusions are obtained:

1. Changes in the roof and bottom displacements slow down due to the backfill. The surface subsidence is reduced and the ground pressure activity is effectively controlled.
2. The overall trend of the roof displacement is down while the bottom is upward. In the excavation and filling processes, the step-displacement relationship shows multi-term variations.
3. The similar simulation experiment method is more conservative than the numerical simulations.
4. The complex geological conditions in the mining area and the assumptions for the modeling process make it difficult to simulate the stress and displacement fields in mining processes using numerical analyses. Therefore, effective on-site monitoring tools should be used to dynamically monitor mining in practical engineering to further optimize the scheme and ensure safe production.

## Supporting information

S1 File (DOCX)
