# Supplementary material for: Simulations of goaf surface subsidence via filling control
Source: PLoS One. 2022 Jan 21;17(1):e0261740. doi: 10.1371/journal.pone.0261740 (PMC8782490; doi:10.1371/journal.pone.0261740)
Supplement: S1 File — (DOCX) [file pone.0261740.s001.docx]

Captions for Supporting Information

We have uploaded the data to Dryad Digital Repository. The sharing link URL of the data set file is as follows:

<https://datadryad.org/stash/share/X6_yVALfjrpGoNdPbomOSWsF5xgf_gzERZSp-U7U2ao>

<https://doi.org/10.5061/dryad.6wwpzgn0r>
